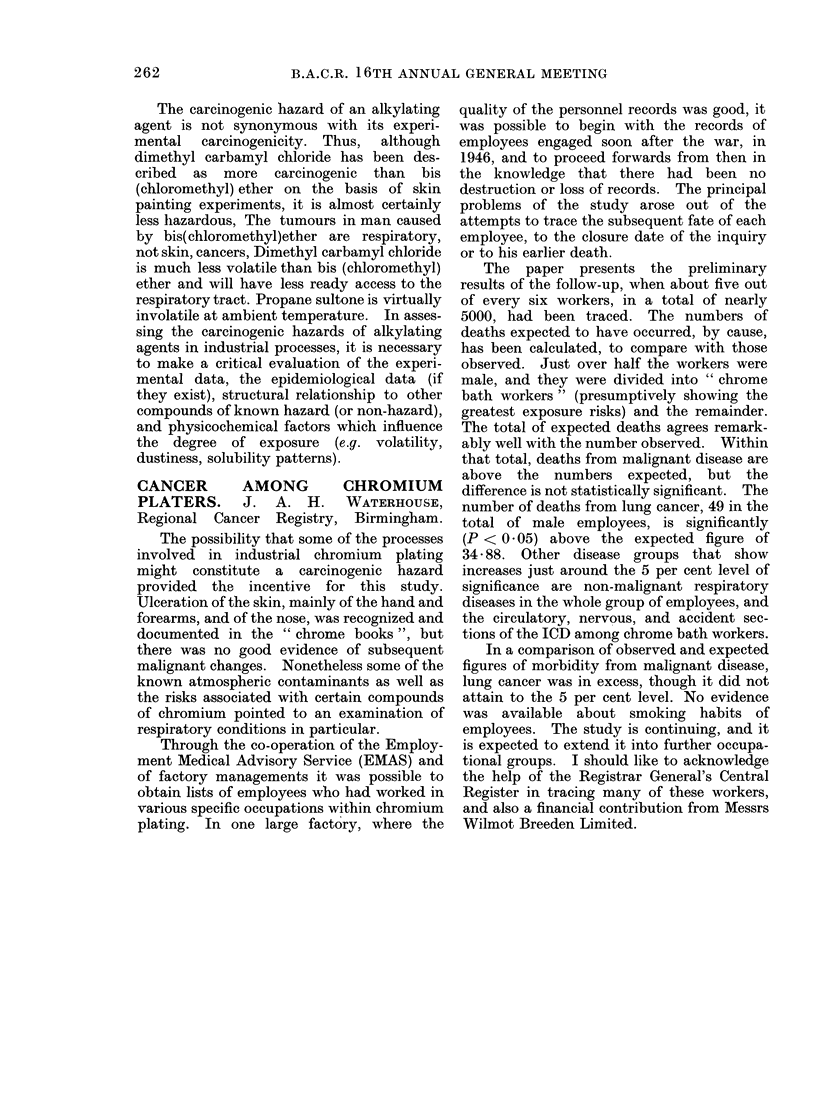# Proceedings: Cancer among chromium platers.

**DOI:** 10.1038/bjc.1975.211

**Published:** 1975-08

**Authors:** J. A. Waterhouse


**CANCER AMONG CHROMIUM PLATERS.** J. A. H. Waterhouse, Regional Cancer Registry, Birmingham.

The possibility that some of the processes involved in industrial chromium plating might constitute a carcinogenic hazard provided the incentive for this study. Ulceration of the skin, mainly of the hand and forearms, and of the nose, was recognized and documented in the " chrome books ", but there was no good evidence of subsequent malignant changes. Nonetheless some of the known atmospheric contaminants as well as the risks associated with certain compounds of chromium pointed to an examination of respiratory conditions in particular.

Through the co-operation of the Employment Medical Advisory Service (EMAS) and of factory managements it was possible to obtain lists of employees who had worked in various specific occupations within chromium plating. In one large factory, where the quality of the personnel records was good, it was possible to begin with the records of employees engaged soon after the war, in 1946, and to proceed forwards from then in the knowledge that there had been no destruction or loss of records. The principal problems of the study arose out of the attempts to trace the subsequent fate of each employee, to the closure date of the inquiry or to his earlier death.

The paper presents the preliminary results of the follow-up, when about five out of every six workers, in a total of nearly 5000, had been traced. The numbers of deaths expected to have occurred, by cause, has been calculated, to compare with those observed. Just over half the workers were male, and they were divided into " chrome bath workers " (presumptively showing the greatest exposure risks) and the remainder. The total of expected deaths agrees remarkably well with the number observed. Within that total, deaths from malignant disease are above the numbers expected, but the difference is not statistically significant. The number of deaths from lung cancer, 49 in the total of male employees, is significantly ($P < 0{\cdot}05$) above the expected figure of 34·88. Other disease groups that show increases just around the 5 per cent level of significance are non-malignant respiratory diseases in the whole group of employees, and the circulatory, nervous, and accident sections of the ICD among chrome bath workers.

In a comparison of observed and expected figures of morbidity from malignant disease, lung cancer was in excess, though it did not attain to the 5 per cent level. No evidence was available about smoking habits of employees. The study is continuing, and it is expected to extend it into further occupational groups. I should like to acknowledge the help of the Registrar General's Central Register in tracing many of these workers, and also a financial contribution from Messrs Wilmot Breeden Limited.